# Piezoelectric Effect of k-Carrageenan as a Tool for Force Sensor

**DOI:** 10.3390/s25154594

**Published:** 2025-07-24

**Authors:** Vytautas Bučinskas, Uldis Žaimis, Dainius Udris, Jūratė Jolanta Petronienė, Andrius Dzedzickis

**Affiliations:** 1 Department of Mechatronics, Robotics and Digital Manufacturing, Vilnius Gediminas Technical University, Plytinės g. 25, LT-10105 Vilnius, Lithuania; vytautas.bucinskas@vilniustech.lt (V.B.); jurate-jolanta.petroniene@vilniustech.lt (J.J.P.); 2 RTU Liepaja Academy, Riga Technical University, Lielā iela 14, LV-3401 Liepāja, Latvia; uldis.zaimis@rtu.lv; 3 Department of Electrical Engineering, Vilnius Gediminas Technical University, Plytinės g. 25, LT-10105 Vilnius, Lithuania; dainius.udris@vilniustech.lt

**Keywords:** force sensor, carrageenan, α-iron (III) oxide, piezoelectric, piezoresistivity, piezoeffect, ferroelectric

## Abstract

Natural polymers, polysaccharides, demonstrate piezoelectric behavior suitable for force sensor manufacturing. Carrageenan hydrogel film with α-iron oxide particles can act as a piezoelectric polysaccharide-based force sensor. The mechanical impact on the hydrogel caused by a falling ball shows the impact response time, which is measured in milliseconds. Repeating several experiments in a row shows the dynamics of fatigue, which does not reduce the speed of response to impact. Through the practical experiments, we sought to demonstrate how theoretical knowledge describes the hydrogel we elaborated, which works as a piezoelectric material. In addition to the theoretical basis, which includes the operation of the metal and metal oxide contact junction, the interaction between the metal oxide and the hydrogel surfaces, the paper presents the practical application of this knowledge to the complex hydrogel film. The simple calculations presented in this paper are intended to predict the hydrogel film’s characteristics and explain the results obtained during practical experiments. Carrageenan, as a low-cost and already widely used polysaccharide in various industries, is suitable for the production of low-cost force sensors in combination with iron oxide.

## 1. Introduction

Over thousands of years, plants and algae have developed structures and special molecules that help them withstand harsh environmental conditions. These flow movements can break or injure the structures of organisms and thus cause their death. The hydrogel-based devices face certain properties of this type of material: diffusion in the hydrogel, swelling forces. The hydrogels become attractive because of their response as a transducer in chemical microsensors, can act as a semiconductor, or work as a pressure sensor due to their cross-linked networks [1]. However, hydrogel-based sensors have low sensitivity, poor stability, poor electrical conductivity, and fillers’ movement within the hydrogel network. The hydrogels ensure significant ion migration for electric current media due to the large water phase in them [2]. The conductive network in traditional piezoresistive sensors under external force develops some cracks that become permanent, resulting in a decrease in the sensing mechanism and durability [3]. Some hydrogel-type material can demonstrate self-healing; in other cases, microcracks are purposefully inserted in advance during the manufacturing process, as a method for increasing the sensitivity of sensors with a suitable Young’s modulus [4]. The hydrogel viscosity mechanisms include adhesion, diffusion, hydrogen bonding, van der Waals forces, ionic, and covalent bonding [5]. However, the general lack of charge carriers limits their conductivity and sensitivity of a sensor [5]. This problem is solved as usual by introducing conductive nanofillers, but the strain sensors still have poor stability due to the movement of nanofillers inside the hydrogel when it is stretched [6]. Hence, the resistance of the conductive material increases with elongation and decreases with a smaller cross-sectional area of the cross-section [7]. Modern science, which has set the tasks of sustainable biodegradation, is learning more and more carefully from nature’s creations.

Carrageenan (Crg) is a natural product that forms the seaweed cell wall, a flexible helical structure molecule with one sulfate group per disaccharide group. In combination with other substances, it forms a seaweed structure that protects the entire organism from damage to the structure and also performs the work of transporting substances in cells. Crg is a polysaccharide, poly-galactan, linearly structured high-molecular-weight anionic sulfate, with many attractive properties for industries [8]. The Crg belongs to the group of multifunctional hydrogels [9]. The Crg is applied in the food industry as a thickener of the supply material of freshness monitoring used to make packaging, electrostatic force sensors, biosensors, biomedical applications, health monitoring processes elaborated for bone tissue engineering or wound healing, and vaccines. The weakest property of k-carrageen (k-Crg) is low water-holding capacity, in comparison with other hydrogels, but it is available for 4D printing [10]. In general, hydrogels can rapidly absorb large amounts of water molecules and remain stable in their applied form and macroscopic structure until they are under certain pressure [11]. Various technologies are applied to enhance the strength of the k-Crg film [12]. Depending on the characteristics of the cross-links, hydrogels can be produced using physical methods such as chain entanglement [13]. One of the methods is mixing with microstructures, such as cellulose microparticles or nanocrystals [14]. Hydrogels enriched with conductive or semiconductive material demonstrate better sensor-related properties. Compression of the metal oxide particles causes local shearing of the oxide films, resulting in potential metal-to-metal electrical paths with relatively low electrical resistance. The descaling effect that occurs during the rearrangement of particles is of significant importance to warrant consideration. The Fe_2_O_3_ represents piezoelectric properties [15] that demonstrate spontaneous polarization that can be reversed by an external electric field [16], named ferroelectricity. The fundamental problem in ferroelectricity is the phase transition, where spontaneous polarization appears or disappears, and the thermodynamic theories of Landau, Lifshitz, and Devonshire do not provide a clear explanation for this phenomenon [17].

The piezoelectric effect is the ability of materials to generate an electrical charge in response to mechanical stress [18]. Piezoelectricity is the process in which mechanical deformation causes electric charge to transfer through the material, or dynamic forces are converted into an electrical signal. Natural piezoelectric materials are cellulose, bones, collagen fibrils, sugars, and peptides [19]. These materials have a unique crystal structure, spontaneous piezoelectricity, are renewable as a resource, have good bio-compatibility, and low cost. Polysaccharides are also a large class of materials and have a successful application in the energy harvesting industry, human–machine interaction [6], piezoelectric energy generators [20], and as a force or pressure sensor with a self-powered function [21]. The piezoelectric effect can be described by the piezoelectric coefficient and piezoelectric constant [22]. Compared to strain gauges, piezoelectric sensors have the best signal-to-noise ratio and the best rejection of high-frequency noise [22]. Mechanical fields and mobile charges in piezoelectric material can interact, resulting in the acoustoelectric effect [23]. In electronically conductive materials, conduction is jointly determined by free charge carriers, such as electrons and holes in semiconductors, or protons in electrochemical charge transfer, and conductive paths. The change in resistance that occurs during material deformation is known as piezoresistivity. During mechanical stimulus, the conductive paths and, thus, the conductivity change, which is the basic principle of the operation of piezoresistive flexible force sensors [24].

Piezoelectric materials can be divided into single-crystal, polycrystalline, polymers, ferroelectrics, and composites. Piezoelectric materials convert electrical energy into mechanical energy or vice versa. Piezoelectric polymers, or the class of “smart materials”, are applied for shock gauges, hydrophones, piezo-cables, for measuring heat [17], for digital systems, or as a smart pavement material with carbon nanotubes [25], carbon nanofiber [26], and graphite [27]. The application fields depend on the size of filler particles [28] and fillers’ capability to dissociate [29].

Some aspects explain the mechanisms of piezoresistive action based on theories of conduction path and tunnelling effect; models describing the piezoresistive properties of material composites are developed by the L. Zhang group [27]. Also, the authors stated that the load rate has little influence on the piezoresistive properties of the material. Q. Zhou [30] declared the improvement of piezoresistivity by coupling piezoelectricity. Ordinarily, the force-sensing range of piezoresistive and piezoelectric sensors is in different ranges when the sensor is manufactured using sandwiched layers of material [31]. In addition to the theoretical basis, which includes the operation of the metal and metal oxide contact junction and the interaction between the metal oxide and the hydrogel surfaces, the paper presents the practical application of this knowledge to the complex hydrogel film. The simple calculations presented in this paper are intended to predict the hydrogel film’s characteristics and explain the results obtained during practical experiments.

The discharge time constant shows the time required for the induced charge to decrease. When the discharge time is long enough, quasistatic forces can be registered with some limitations due to current leakage [32], leading to the measurement of only dynamic forces.

Polymer-based electronics have some weak points: operating frequencies, low touch registering rate, thermal stability, and chemical resistance [33]. Flexible polymer-based sensors manufactured from polyimide, polyester are only flexible, and other properties are represented by additives. The internal structures of polymers respond to mechanical load by the molecular segments’ motion [33].

The main aim of this work is to analyze the direct piezoelectric effect of biodegradable k-Crg film, caused by the mechanical impact, and measure energy transformation efficiency. In addition, this confirms the theory of charge transfer in k-Crg coupled with iron (III) oxide microparticles in hydrogels.

## 2. Materials and Methods

All the chemicals (isopropanol, α-Fe_2_O_3_, other metal oxides, and glycerol) were purchased from Sigma-Aldrich (Taufkirchen, Germany), except the k-Crg, which was delivered from Liepaja University, Latvia, and was prepared according to this lab-developed methodology [34], where preparation stages can be divided into physical and chemical processes. The selected k-Crg liquid contains some plant moieties, including cellulose molecules working as a static charge generator. All the supporting materials, such as aluminum foil and transparent plastic plates, were obtained according to the description of the internal procedures of the laboratories. All liquids were prepared in deionized water of 0.5 µS/cm conductivity. The pH of mixed dispersions was measured using a digital pH meter (Mettler Toledo GmbH, Greifensee, Switzerland). The conductivity was measured using a DIST 3 Conductivity Meter (Hanna Instruments Baltics, Vilnius, Lithuania). The capacitance was measured by UT 603 meter (Uni-Trend Technology Co., Ltd., Dongguan, China). The size of the α-iron (III) oxide particles distribution and specific surface of α-iron (III) oxide particles were calculated to include 0.1 g of α-iron (III) oxide particles by using Fritsch Analysette 22 software (Fritsch GmbH, Weimar, Germany).

The k-Crg samples were prepared according to the protocols of previous experiments [35]. To assess the influence of plastic deformation on the reliability of the results, samples with the same material composition but different thicknesses were selected: 1 mm, 2 mm, 4 mm, and 8 mm. To apply a constant force, a steel ball weighing 7 g and a cylinder weighing 18 g were used. The different shapes of bodies affect the hydrogel in different ways.

The material homogeneity and film sensitivity to mechanical load were investigated using a custom-made micro-robotic system, which applied controlled compressive forces at selected points on the film surface. The mechanical and piezoresistive properties of the prepared material samples were investigated by a computer-controlled tension–compression machine, Mecmesim MultiTest 2.5-i (Mecmesim Limited, Slinfold, West Sussex, UK), with a maximum load of 25 N, coupled with a UNI-T UT55 Multimeter (Uni-Trend Technology Co., Ltd., Dongguan, China) for synchronous recording of resistance under controlled force and displacement to evaluate the hysteresis effect and plastic deformation.

For material surface conductivity measurements, we constructed an experimental setup of a 3D-printed plastic holder for k-Crg film and 4 Al foil upper electrodes with a 5 mm × 10 mm contact area. Upper electrodes were placed near each edge of the rectangular-shaped sensitive material, maintaining an equal distance between the electrodes on opposite sides. A ground electrode, serving as the cathode, was placed under the film over the entire area. During the experiment, this setup was connected to the personal computer through a data acquisition system and a voltage divider circuit.

The measurement was performed by pressing the film with a controllable force on a 10 mm^2^ contact area, using a cylindrical plastic dielectric rod. The potential difference between the four electrodes and the ground electrode was registered by the device National Instruments USB-4432, 5 channel (NI, Austin, TX, USA). The pressure force was additionally controlled by placing the balance under the holder with the k-Crg film. The measurement accuracy fits within ±2%.

**Figure 1:**
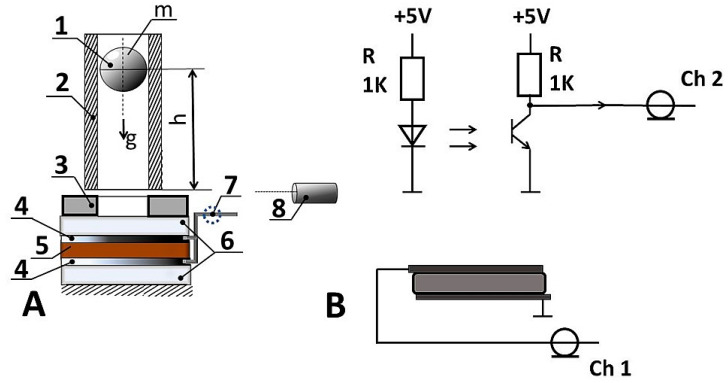
Visual representation of experimental setup: (**A**) description of experimental bench, where 1—metal ball; 2—plastic tube; 3—movement sensor; 4—alumina electrodes; 5—k-carrageenan and α-iron (III) oxide film; 6—lamination of film; 7—shielded cable; 8—optical sensor and (**B**) principal scheme of experimental setup.

The main measurements of the response to the dynamic impact were made using a Hantek DSO 6082 BE digital oscilloscope (Qingdao Hantek Electronic Co., Ltd., Qingdao, China), with two independent channels with a bandwidth of 80 MHz and sampling rate up to 250 MS/s. The single measurement results are saved in a CSV file, with 10,240 samples for each screen, consisting of 10 divisions on the time axis, and measurements ranged from 2 to 20 ms. The first channel of the oscilloscope registered the potential difference between the upper and lower electrodes (Figure 1) during the hit of a metal ball or cylinder on the material surface. To prevent current leakage and maintain a constant contact area and compressive force between the electrodes and the film surface, the k-Crg film with electrodes was sealed using parafilm and mounted on the experimental table. The contact between the film and the falling ball/cylinder was monitored by a photosensor installed in the tube 3 mm above the surface of the desk. The measurements were made in single-pulse mode with a threshold trigger value of 16.3 mV. The oscilloscope was preferred due to its ability to register rapid voltage changes during a short performance time. In order to minimize hit signal delay, an optical sensor was installed inside the tube, in which the ball or cylinder is thrown. The workplace is represented in Figure 1. Channel 1 of the oscilloscope records the potential changes caused by the impact of the falling ball on the surface of the force-sensitive film, and channel 2 synchronously records the signal from the barrier optical sensor, indicating the contact of the ball with the surface. In this way, the system could evaluate the speed of the k-Crg film reaction to the impact. The electrodes were placed on both film sides to ensure appropriate charge transfer. Before and after the experiments, the quality of the k-Crg film was evaluated optically and mechanically. The result of the impact of the ball and flat cylinder gives a pressure difference in the affected area, causing a restoring force and deformation along the perimeter of the film [36]. Von Karman’s study showed that the pressure on the surface during impact can rise significantly in the case of a flat active surface [37]. Thus, this theory can illustrate the processes of force impact on the hydrogel. The water content in the hydrogel is in different states: strongly bound, weakly bound to a biopolymer network, and non-bound water molecules. Depending on environmental conditions’ changes, the proportion of bounded and non-bounded molecules is changing with very fast conformational rearrangements on the picosecond scale [38].

All experiments were carried out after removing possible extraneous effects: vibration, resonance of metal parts, the possibility of friction of the ball against the tube, suitable contact wires, and metals were selected. Data reproducibility was assessed by performing replicate experiments.

### Characterization of the Material

Actual calculations to explain measured potential changes between two electrodes attached to the k-Crg film during mechanical hit and applied formulas are represented in the text below.

The gauge factor (GF) is the most important parameter to evaluate force sensors. The calculation is performed by Formula (1), where resistance *R*_0_ is the initial resistance of the sample, *L*_0_ is the initial length of the hydrogel, and *R* (Ω) and *L* (cm) are the resistance and length of the hydrogel at the point of stretching, respectively.

(1) $$GF=\frac{\frac{R-R_{0}}{R_{0}}}{\frac{L-L_{0}}{L_{0}}}$$

The *GF*, the sensitivity of a sensor, was obtained by linear fitting the change curve of the relative resistance.

The conductivity (*σ*, mS/cm) of the k-Crg stretchable film samples is calculated by Formula (2).

(2) $$\sigma =\frac{L}{RS}$$

where *σ* (S m^−1^) is the conductivity, *S* (m^2^) is the cross-sectional area, and *L* (m) is the distance between the two electrodes [39].

Piezoelectricity is an effect directly proportional to the material permittivity, the electric field strength, the elasticity/flexibility of the material, and the applied stress. Important values to describe piezoelectricity are the stress/strain vector and the piezoelectric constant. The piezoelectric equation, according to other authors, can be expressed as follows [18,19,40]:

(3) $$D_{k}=d_{k,i,j}T_{i,j}$$

(4) $$S_{i,j}=d_{k,i,j}E_{k}$$

where *D_k_* (Cm^−2^) is electric displacement, *T_ij_* (Nm^−2^) is stress component, S*_ij_* is strain component, *E_i_* (Vm^−1^) is the electric field component, and *d* is the component of piezoelectric charge or strain constant, when *i*, *j*, *k* = 1, 2, 3 are the material coordinates.

The piezoelectric charge coefficient depends on the electric charge per unit area under applied mechanical force [41].

Practically measured capacitance of the sample can be expressed by the formula:

(5) $$C=\frac{\varepsilon_{r}\varepsilon_{0}A\;}{t}$$

where $\varepsilon_{r}$ is the relative permittivity, $\varepsilon_{0}$ the vacuum permittivity, *A* the active electrode area, and *t* the thickness. The relative permittivity of the material is calculated using Formula (5).

The piezoelectric coefficient *d* is calculated using the formula:

(6) $$d=\frac{\varepsilon_{r}\varepsilon_{0}AV}{Ft}$$

where *V* is the voltage, *F* the force, *t* the thickness, $\varepsilon_{r}$ the relative permittivity, $\varepsilon_{0}$ the vacuum permittivity, and *A*, the active electrode area.

When a polymer is amorphous, piezoelectric behavior can be explained by molecular dipoles. In the k-Crg case, the sulfate groups play an important role.

The iron oxide, n-type semiconductor [42], is essentially not a homogeneous structure and is mostly dominated by cation vacancies on the surface, and this provides a context for understanding the iron oxide surface with redox processes [43]. The iron diffuses into and out of the bulk in response to the chemical potential of oxygen, forming sometimes complex intermediate phases at the surface of the oxide particle. The charge transfer in iron (III) oxides is complex, as it is in the iron (III) oxide water/liquid interface [44], although this oxide is considered to have poor electrical conductivity.

Another charge transfer method in hydrogels is small liquid gaps surrounded by hydrogel molecules with sulfate groups, ensuring the electrochemical charge transport, or more precisely, positive charge transport in these liquid gaps. In this case, resistivity can be described by Formula (5):

(7) $$R_{i}=\left(n_{i\;}-1\right)R_{matrix\;}+n_{i\;}R_{fillers}$$

where $R_{i}$ is the resistance of every conductive path between particles in the matrix (hydrogel), $n_{i}$ is the number of fillers in each conductive path, $R_{matrix}$ is the resistance of the hydrogel between the adjusted fillers, and $R_{fillers}$ is the resistance of filler (iron (III) oxide). When the filler particles are very small, a simple formula is valid if the resistance of all enabled conductive paths $R_{i}$ is directly proportional to the number of conductive fillers and the resistance of the matrix. The iron oxide particles were about 1 mm in size in this experiment. The density of iron oxide particles was approximately 5.2 g/cm^3^. After estimating the density of iron oxide particles and their surface area, the 1 cm^3^ of prepared k-Crg hydrogel consists of approximately 3 cm^3^ of a specific area of iron oxide particles, which can participate in negative charge transfer. In the case of the ideal impact of a ball on a hard surface, the velocity after impact $\nu_{f}$ should be the same magnitude as its velocity before impact $\nu_{i}$, because of unchanged kinetic energy [36]. During inelastic impact, when energy losses occur, the impact can be explained according to the restitution coefficient **e**, which is directly proportional to $\nu_{f}$ and inversely proportional to $\nu_{i}$. The potential energy in the ball impact case works according to Hook’s law. It is known that the stress–strain behavior depends on the strain rate [45]. In a real ball hitting the surface, the energy is dissipated due to the collision. The coefficient of restitution describes the elementary mechanics of ball drop [46]. The impact model can be described as kinematic (Newton), kinetic (Poisson), or energetic (Stronge). Numerous researchers have developed impact models, including plastic and viscoelastic models: Maxwell, Kelvin–Voigt, Yigit and Christoforou Johnson, etc. [46]. Although the simulations are easier to describe the impact, the accuracy of numerical analysis leads from theories to practical investigations, and this is the reason for developing and modifying impact models today.

When the strain is low, the material may have a lower elastic modulus, and with a high strain rate, the material can demonstrate a harder response to deformation. This nonlinearity can be attributed to the material’s crystal structure, dislocations, and inhomogeneity.

**Figure 2:**
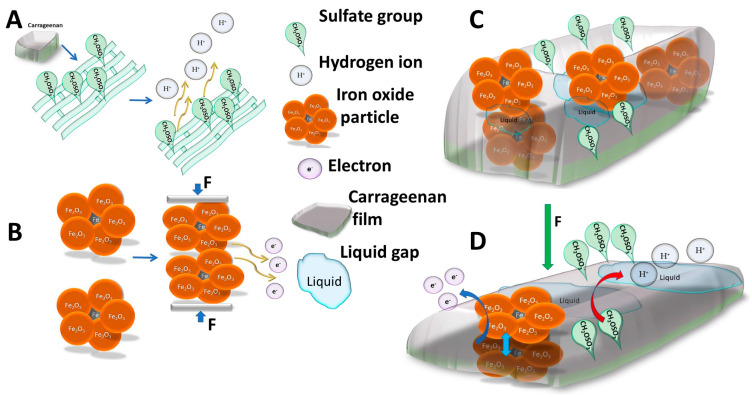
Visual representation of charge transfer of k-carrageenan film with iron (III) oxide particles during short-term force hit. (**A**) k-Carrageenan film electrochemical reaction to the applied force: the protonic charge transfer proportional to applied force; (**B**) iron (III) oxide semi-conductor charge transfer which initiates the particles under pressure; (**C**) visual representation of prepared film main parts participating in charge transfer: non-uniformly distributed iron (III) oxide particles, liquid gaps, carrageenan sulfate groups; (**D**) emitted electrons, released hydrogen ions, and liquid gaps acting as part of an electrochemical microcell guaranteeing charge transfer.

The k-Crg film with spontaneous resistance fluctuations led to assumptions that this polysaccharide corresponds to the character of a piezoelectric material. The complex charge transfer during applied force can be explained by the nature of the polysaccharide and semiconductor combination. The k-Crg is a conductive polymer host in the electrolyte system to be involved in oxidation/reduction reactions [47]. The literature-based graphical representation of possible charge transfer during mechanical load in k-Crg/Fe_2_O_3_ film, based on published articles, is presented in Figure 2.

The k-Crg film is known as a material sensitive to pressure, demonstrates ionic charge transfer, and corresponds to the class of polysaccharides as piezoelectric materials. Iron (III) oxide also participates in charge transfer. In the presence of oxygen vacancies, α-Fe_2_O_3_ is generally an n-type semiconductor, but with some doping, it can work as a p-type semiconductor due to a narrow band gap. The solid phase, iron (III) oxide, is in contact with k-Crg bio-polymer, droplets of initial solution residues, and air gaps. The contact point changes during applied force (compression) and generates micro-cracks, which fill with the fluid from surrounding gaps, or due to simple deformation of k-Crg polymer when increased surface area creates better conditions for sulfate groups to participate in charge transfer. Here, we report an ionic piezoelectric hydrogel film capable of acting as a nanogenerator that enables biomechanical energy harvesting and touch or impact. The tensile (strain 10%) and compression described in previous articles are available upon request. This study presents a stretchable, biocompatible, environmentally stable energy harvester and touch sensor, with potential applications in smart artificial skins, soft robots, functional displays, and wearable electronics.

## 3. Results and Discussion

### 3.1. Piezoresistive and Surface Conductivity and Visual Investigation of k-Carrageenan Film

**Figure 3:**
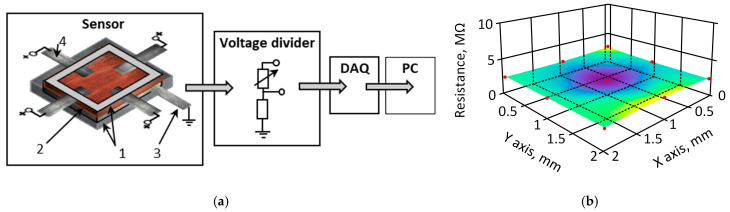
Representation of distribution measured resistance on surface area on applied constant force with step 1 mm. (**a**) Scheme of experiment setup for k-Crg film surface electrical resistance investigation. Sensor layers: 1—plastic holder, 2—sensor film, 3—cathode or ground electrode, 4—metal (+5 V) electrodes; voltage divider; data acquisition system; personal computer. According to [48], (**b**) surface area of measured resistance distribution during applied force (pressing) to the surface.

The initial experiment was conducted to assess the quality of the film in terms of its ability to respond evenly to applied force across the whole surface of the sample. To perform this measurement, a controlled compressive force was applied to the film surface, and the electrical resistance difference between the electrodes was measured. For this measurement, the 1 mm-thick k-Crg film was placed into a plastic holder. One electrode, the size of which was equal to the film, was placed under the sensitive k-Cr film. Four electrodes were attached to the edges of the film surface at an equal distance from each other. Results are represented as a surface area electrical resistance map. The distribution of measured resistivity on the film area by applying a constant force in selected points is represented in Figure 3. The measured resistance (Figure 3) values ranged from 3.5 MΩ to 3.9 MΩ when the surface area was 50 mm^2^ and the electrodes were placed on the sample border lines with a contact area of 5 mm^2^ for every electrode on top of the sample.

**Figure 4:**
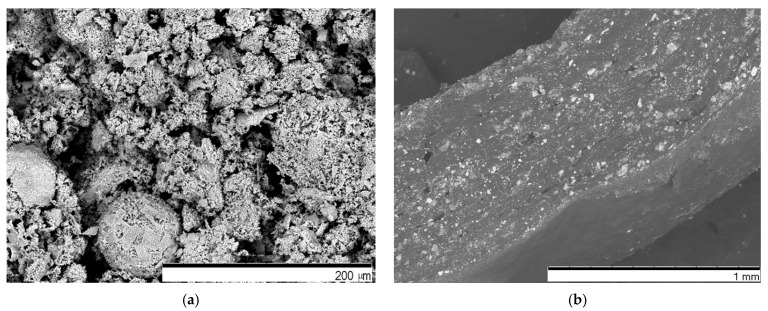
The iron (III) oxide particle surface representation and k-Carrageenan film with iron oxide structure: (**a**) pure iron oxide particles, 15.0 kV, Mag = 100; (**b**) the cross-section of k-Crg film.

As shown in Figure 3, the dependence of measured electrical resistance on the distance between the electrodes corresponds to the classical theory of electrical conductivity. The area of lower measured electrical resistance in the center of the conductive film corresponds to the distance between electrodes. The k-Crg film for this experiment was 1 mm thick, containing 1.8% of iron (III) oxide particles with a size of about 1 µm, with a conductivity of 10^−14^ S/cm [49], and 1.6% of glycerol as a plasticizer. The concentration of plasticizer is crucial for k-Crg film. Without the plasticizer, the film lost sensor-relevant properties in 168 h, and with the plasticizer, all physical properties were maintained for more than 6 months. The liquid k-Crg electrical conductivity was 0.27 ± 0.01 m S/cm, the electrical resistance of the liquid was 0.04 ± 0.01 MΩ, and after the addition of iron (III) oxide, it was 0.08 ± 0.02 MΩ. When the sample was formed into the film and dried, the resistance increased to 1.2 ± 0.1 MΩ. The film’s electrical resistance depends on the iron (III) oxide concentration in the sample and varies from 0.4 MΩ to 13 MΩ. Hence, we selected film sensitivity 0.35 ± 0.2 MΩ N^−1^, GF = 10.4. The electrodes, made from aluminum foil, were 10 mm × 100 mm × 0.2 mm in size. Recorded relaxation time is less than 40 s, and the fitting curve for the relaxation curve corresponds to a polynomial order of 2. The surface structure and the cross-section investigation were performed to evaluate the qualitative parameters of the piezoresistive effect of the k-Crg film. The film’s structure is used to explain the mechanism of the piezoelectric response to mechanical stress. Since the initial mass for flexible skin-like film was a liquid, it acquired a micro-wrinkled surface during the drying process. This surface relief likely provides protection against micro-cracks. The iron oxide (III) particles are not exposed on the prepared film surface, due to natural differences in material density. By analyzing the particle size distribution by Powder Analyzer, the particles of iron (III) oxide were mostly about 1 μm, about 81% of all particles, and correspond to a Gaussian distribution. In Figure 4, a micrograph of iron (III) oxide particles with a huge surface area is represented.

Figure 4b represents the cross-section of the sample, where an inhomogeneous structure is established. There are different-sized iron (III) oxide particles surrounded by initial liquid moieties, air gaps, and k-Crg biopolymer. The Fe_2_O_3_ particles (Figure 4b) in the bulk film are distributed unevenly, even though a heating magnetic stirrer was used during film preparation; probably, the gelatinization process should be accelerated. Despite this uneven distribution of materials, the product acts as a force film. In Figure 4b, the contact of several phases is represented, which has a special influence on the charge transfer during mechanical load. If the phase contact formed between the iron (III) oxide particles and biopolymer is broken during the mechanical load, this will be eliminated by droplets of the liquid, which contains isopropanol, an electrolyte to ensure proton conductivity. The air gaps in the films during applied force guarantee internal micro-cracks where the liquid spreads from gaps, thus further increasing the surface area where charge transfer can take place. This may be one of the reasons for the increase in potential during the mechanical hit. However, smaller particles are arranged around the spherical particles. Thus, in our experiment, it is necessary to take into account the influence of piezoresistivity and piezoelectric effect on different-sized iron (III) oxide particles, including some of nano-size. The mechanism of charge transfer during applied force can be explained due to the strong negative charge of k-Crg, containing a sulfate group, and exhibits a high capacity to react with positively charged substances.

**Figure 5:**
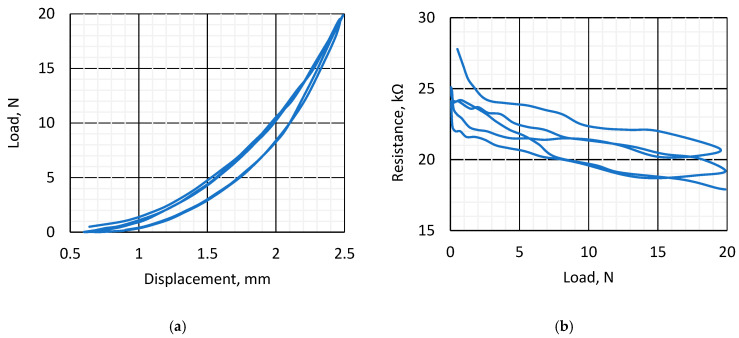
Mechanical load dependence on displacement and resistance of the sample. (**a**) Stress–strain curves. Cyclic applied force pressure curve of the hydrogel film. (**b**) Dependence of measured resistance on applied cyclic mechanical load.

In Figure 5a, the hydrogel film’s response to mechanical load is represented, where 3 cycles of measurement are represented. The measurements were performed simultaneously using a mechanical force bench and a resistance measurement set. The resistance values in the first cycle are different from the subsequent ones, which is typical for most materials (Figure 5b). In the subsequent cycles, the hysteresis loops almost coincide. Obviously, at lower loads, the scatter in the resistance data resulted from deformation-influenced contact area between the electrode and the hydrogel. Figure 5b represents the dependence of the measured resistance of the k-Crg sample on the applied pressure on the whole surface. At the beginning of the experiment, the measured resistance was 27.8 kΩ at zero mechanical load, and it decreased with each cycle. The apparent resistance drifts can be attributed to problems in the contact between the metal electrode and the gel due to the different flexibility of these materials under force. The “ripples” observed during the cycle can be attributed to the redistribution of the gel’s internal structure resulting from compression. Resistance values are also influenced by the compression of iron particles and the friction between them. A drift in resistance values at the end points of the cyclic curve is also observed, which corresponds to material plastic deformation typical for hydrogels.

### 3.2. Investigation of Piezoelectric Behavior of k-Carrageenan Film

**Figure 6:**
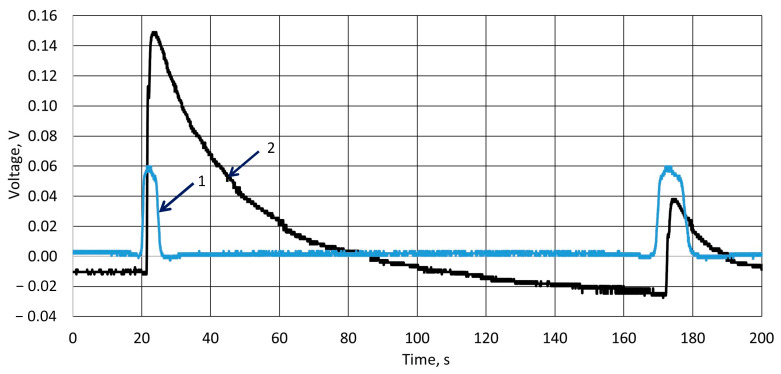
Registered impact on the surface of a 1 mm-thick hydrogel film force sensor by a metal ball dropped from 0.15 m high. 1—measured potential of Crg film; 2—registered moment of ball contact with surface by optical sensor.

In Figure 6, the initial experimental results, which define the piezoelectric behavior, are presented. As we can see from the measurement results, from the channel 1 and channel 2 curves, the recorded impact of the ball on the film and the measured potential growth started simultaneously. Where channel 1 represents influence on potential values during mechanical hit of a falling ball (7 g) on the k-Crg film (20 mm × 20 mm × 1 mm), and channel 2 represents the control system which reacts to the optically registered fact of ball contact with the surface of the film. The potential reaches its maximum value in about 3 ms.

Comparing both signals, it is seen that the peak in the optical sensor signal appears first, and the response of the sensor shows some delay; however, it is a result of the offset between the beam of the optical sensor and the material surface; in our case, it was equal to 2.8 mm. Therefore, this delay is taken into account and does not impact the further provided results. The observed next peak in the curve represents the bouncing of the ball from the surface. Similar results were obtained after performing a series of back-to-back tests with a one-minute (60 s) interval between experiments, i.e., a signal delay affecting material fatigue was not observed, nor was it recorded. The data presented in experimental curves were not normalized or otherwise processed, so errors and scatter in voltage values are visible both at the beginning of the experiment, when the potential difference zero should be recorded, and in the further stages of the experiment. This shows that the data reproducibility is very good, despite the fact that the biopolymer must experience the fatigue that is a conventional characteristic of such materials.

**Figure 7:**
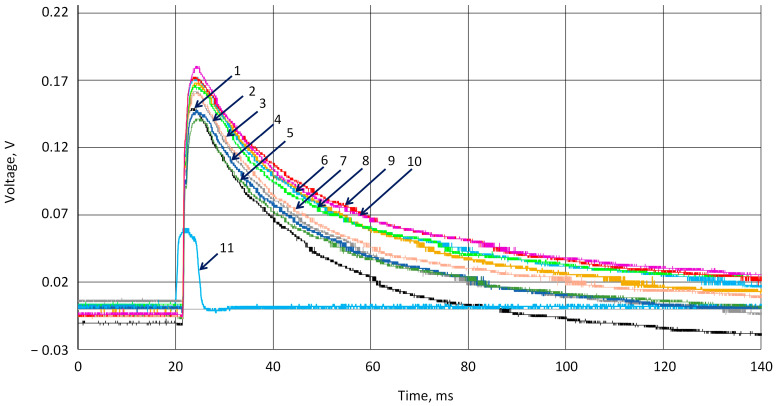
Registered series of impact on the 1 mm-thick hydrogel surface of the force sensor by a metal ball, dropped from 0.15 m high. Curves 1–10 film response to 10 hit cycles. 11, signal from optical sensor.

**Figure 8:**
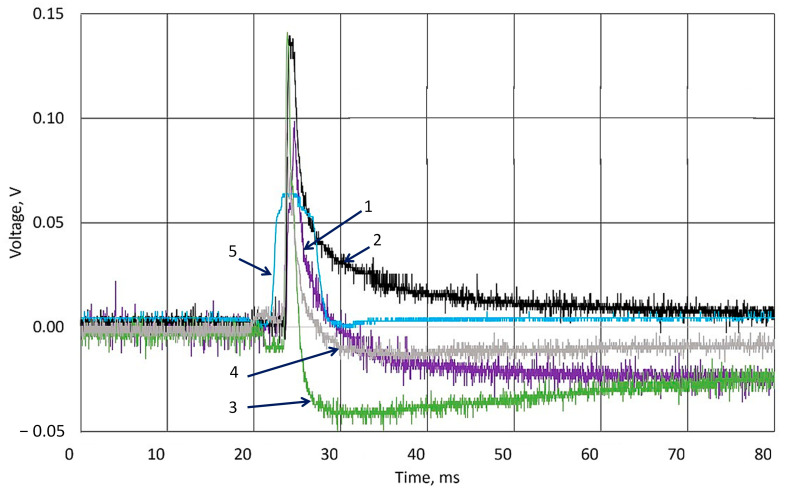
Experimental results of generated voltage using body drop from 0.15 m height to 2 mm thickness of k-Crg film. Drop of ball: 1—the 1st experiment; 2—10th experiment; Drop of cylinder: 3—1st experiment; 4—10th experiment; 5—signal from optical sensor.

**Figure 9:**
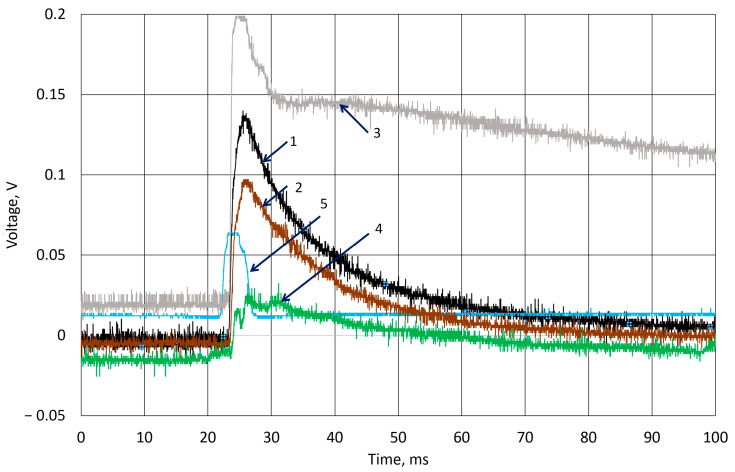
Experimental results of generated voltage using body drop from 0.15 m height to 4 mm thickness of k-Crg film. Drop of ball: 1—the 1st experiment; 2—10th experiment; Drop of cylinder: 3—1st experiment; 4—10th experiment; 5—signal from optical sensor.

As shown in Figure 7, all experiments have similar potential growth after the ball hits the surface. The results were not normalized. Ten experiments were carried out with a 1 min pause. The results are signed in colors and represented in the figure legend. The blue line marked as signal from channel 2 shows the moment of ball contact with the surface. The response to the hit is a delay in registering the signal, which can be attributed to all parts of the signal registering system, including the oscilloscope’s ability to display the received signal on the screen. The difference was registered in the initial potential values. This small drift of the potential (less than 0.1 V) measured between two surfaces of the k-Crg film sample before the hit can be explained by changes in the material’s structure, because the applied force resulted in some damage to the structure of the film. As we can see in Figure 6, the fatigue of the sample is recorded as the fluctuations in the voltage values, but the ability to respond to the applied shock remains unchanged. Considering the film’s heterogeneity and the cheapness of the starting materials, we assumed that these abundant and cheap, environmentally friendly materials should find good practical application in various areas where contact of the sensor material with a person is required. Figure 7, Figure 8 and Figure 9 show the impact on the k-Crg film affected by the ball, resulting in the plastic impact of force and cylinder, resulting in a flat impact of force on the surface. When the impact of the flat causes the liquid to cavitate, cavitation occurs. The k-Crg is a biopolymer created by nature, designed to withstand strong mechanical impact, and has developed a naturally self-healing function. The impact of a cylindrical object on the surface of the k-Crg film demonstrates the fatigue of this material in the simplest way compared with the same investigation using the ball. Ten experiments were performed with a 1 min pause. The figures represent data of the 1st and the 10th experiments.

All k-Crg films were prepared using the same conditions as the previously analyzed film. Figure 8 presents the results of the ball and cylinder drop in the k-Crg film. The moment of the ball hitting the surface of k-Crg generates an electrical signal, and the resulting emf was measured. The line denoted in blue represents the signal from channel 2 of the oscilloscope as a synchronization signal, which is registered by the photosensor during ball contact with the sensor surface. In this experiment, the greatest voltage was registered when the cylinder hit the surface for the first time. The measured voltage was higher during every measurement when the hydrogel sample was hit by a ball and lower when an experiment used a cylindrical body. Hence, the resulting voltage in the first measurement was about 0.1 V; at the 10th measurement, it was similar to the experiment that was performed with a falling ball. As the k-Crg is practically an electrolyte containing a sulfate group, the electrical charge transfer is limited by the electrochemical reaction rate and charge transfer in liquids.

The response of a 4 mm hydrogel film to the hit of a cylindrical object is represented in Figure 9. The 7 g ball impact after 10 experiments measured a potential decrease of less than 0.05 V. The impact of the cylinder causes damage to the material and results in different voltage curves (Figure 8, lines 3, 4).

**Figure 10:**
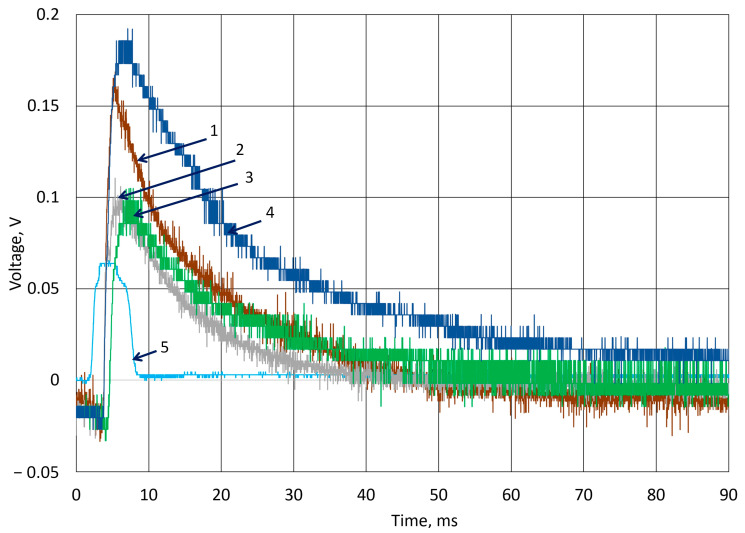
Experimental results of generated voltage using body drop from 0.15 m height to 8 mm thickness of k-Crg film. Drop of ball: 1—the 1st experiment; 2—10th experiment; Drop of cylinder: 3—1st experiment; 4—10th experiment; 5—photosensor reaction to impact.

Figure 10 represents experimental research using an 8 mm-thick sample. The k-Crg film withstands the impact force of the cylinder, as we can see from the curved shape. This sample generates higher noise than previously investigated samples. Since electrodes were placed on both sides of the film, the film of 8 mm thickness better withstands impact, but produces a lower quality signal. According to the measured results, the piezoelectric coefficient of k-Crg with the Fe_2_O_3_ film was calculated. Capacitance measurement was performed for samples of 1 mm, 2 mm, 4 mm, and 8 mm. Calculations of the latter are carried out using Formulas (5) and (6). The calculated piezoelectric coefficient represents its value for a complex hydrogel mixture, containing biodegradable k-Crg with *Furcellaria Lumbricalis* algae skeletal material moieties and particles of Fe_2_O_3_ containing metallic iron atoms. The numerical value of the piezoelectric coefficient of such material was observed to be about 25 pC/N. The ε_r_ for k-Crg/Fe_2_O_3_ composition in our case was (4.11 ± 0.2) 1 × 10^4^, and it depends on the composition of the sample and the biodegradability level of the polysaccharide. Such characteristics define the suitability areas of application of this composition as s film for a biodegradable force sensor. The data in the table are average values calculated from a series of ten experiments. Such statistical data have different scattered values, because the results are influenced by the probable non-homogeneous distribution of components due to natural biopolymer molecules, regardless of the technologies applied for film manufacturing. Also, the results are affected by the fatigue of the material, which depends on the thickness of the film. Overall, the potential difference of 0.2 V to 0.1 V was recorded during applied force, regardless of how the impact was made with the ball of the cylinder. It is obvious that the optimal film thickness for the measurement of force impact must be not less than 2 mm because, in our investigation, an 18 g cylinder damaged the 1 mm k-Crg hydrogel film. As a result, the dropped ball resulted in plastic deformation of the k-Crg film and enabled the material to self-healing after the applied force was removed [50].

## 4. Conclusions

The requirements for force sensors in recent years have been supplemented with several new conditions: biodegradability, low price, environmental and consumer friendliness, and suitability as renewable resources. These and similar requirements force scientists to review the production costs and safety of existing sensors for use and disposal. Materials with piezoelectric properties created by nature find increasingly wider applications as science develops. The natural biopolymers produced in plant organisms change depending on the prevailing environmental conditions. Multifunctional force sensors can be developed by creatively exploiting this variability in the composition of biopolymers. The k-Crg in this study was selected with parts of the plant that not only make the starting raw material cheaper but also increase the piezoelectric properties of the film by elaborating cellulose, but ensure strength to withstand the impact of mechanical force. The structure of the iron oxide particles is no less important. The implemented iron oxide used for the pigment is not completely homogeneous in its composition, which enhances semiconductor properties.

The piezoelectric material is currently the most sought-after material in the manufacturing of wearable sensors, where it is important to use a material with self-generating electrical current ability. The hydrogel film k-Crg and Fe_2_O_3_ investigated by applied mechanical force resulted in a stable voltage, almost independent of the thickness of the film. Mechanical impact of a steel ball, falling from 150 mm, generates a voltage of 0.15 V to 0.17 V under open-circuit conditions. The calculated piezoelectric coefficient depends on the type of force applied by a flat or round object. The self-healing potential of the k-Crg film has been studied in sufficient detail by other authors and also observed during our experiments.

Polysaccharides are recognized as piezoelectric materials, cheap, renewable, human-friendly, and biodegradable piezoelectric materials. The sensitivity of polysaccharides, including k-carrageenan, to temperature, humidity, and similar environmental factors is also a positive property that can be applied to sensors. The electrical behavior, elasticity of the k-Crg biofilm, and other parameters of this polysaccharide, as well as the practical experiment to register mechanical load, rank in the same order of quality as other piezoelectric polymers.

As we can see from the presented studies, the most optimal and least noise-generating film thickness is 1 mm. We conclude that the k-Crg/Fe_2_O_3_ film for the force sensor that has been developed and tested responds well and qualitatively to a single impact. Due to the renewable resources of its starting materials, such a product could be used as a force sensor to detect impact damage to an object. Nevertheless, it is necessary to further focus on improving the electrode film connection and researching possibilities to use alternative conductive materials.

## Data Availability

The original contributions presented in this study are included in the article. Further inquiries can be directed to the corresponding author.
